# Retraction: Interaction between Age and Obesity on Cardiomyocyte Contractile Function: Role of Leptin and Stress Signaling

**DOI:** 10.1371/journal.pone.0271530

**Published:** 2022-07-12

**Authors:** The PLOS ONE Editors

Following the publication and subsequent correction of this article [1, 2], the University of Wyoming informed *PLOS ONE* that during institutional examination of the article, evidence was found of data irregularities and image reuse affecting Figures 5, 7, 8, and the original, uncorrected Figure 2. Specifically:

- The C57-Aging 12mo and the *ob/ob-*aging 12mo panels of the original Figure 2 appear similar if one of the panels is rotated 180°.
- Areas indicating splicing and inappropriate image manipulation were detected in the following panels:
  ◦ Figure 5B *p*^*47phox*^ 47KD Young, and *p*^*47phox*^ 47KD Aging -12mo
  ◦ Figure 7A pAkt Young, pAMPK Young, and AMP Aging -12mo
  ◦ Figure 8A pp38 43KD Young, and pp38 43KD Aging -12mo
  ◦ Figure 8C ERK 42/44KR Aging -12mo

In addition to these concerns, editorial reassessment of the article raised further concerns with results presented in Figures 6, 7, and 8. Specifically,

- In Figure 6A, lanes 2–6 of the GAPDH 37KD Young panel appear similar to lanes 2–6 of the GAPDH 37KD Aging -12mo panel if one of the panels is rotated 180°.
- Additional irregularities were detected in the background of the following panels:
  ◦ Figure 7A peNOS Young
  ◦ Figure 8C pERK 42/44KD Aging -12mo

The corresponding author confirmed that some of the panels in Figures 5, 6, 7, and 8 were prepared using spliced images. They explain that the original submission used an 8-group study design, and that additional groups were added following peer-review. The use of data obtained from spliced blots was not clearly indicated during figure preparation, as was required per the *PLOS ONE* image preparation guidelines in place at the time of submission. Furthermore, the corresponding author confirmed that the GAPDH panel in Figure 6 was duplicated inadvertently.

Although the corresponding author provided underlying data and repeat experiment data in support of their article, the data provided for editorial review were not sufficient to resolve the concerns with the panels presented in these figures.

In light of the concerns affecting multiple figure panels that question the integrity of these data, the *PLOS ONE* Editors retract this article.

JR and JP did not agree with the retraction. LEW agreed with the retraction. FD, GJC, PZ, and JMN either did not respond directly or could not be reached.
